# Working animal welfare and their multidimensional roles on livelihood improvement in Ethiopia: A systematic review and meta-analysis

**DOI:** 10.1017/awf.2024.68

**Published:** 2025-01-07

**Authors:** Getasew Daru Tariku, Tarekegn Derbib Biza, Senait Kehali Tesfaye, Sinkie Alemu Kebede

**Affiliations:** 1 Department of Rural Development and Agricultural Extension, College of Agriculture and Natural Resources, Mekdela Amba University, PO Box 32, Ethiopia; 2 Department of Animal Science, College of Agriculture and Natural Resources, Mekdela Amba University, PO Box 32, Ethiopia

**Keywords:** Animal welfare, Ethiopia, food security, roles, livelihood, working animals

## Abstract

Working animals have a crucial socio-economic role to play for many low-income communities. One such example is in Ethiopia where virtually all the draught power for agricultural production derives from working animals. However, despite this, the welfare status of working animals in this country remains poor. Hence, a clear understanding of the major welfare problems faced by working animals is key to helping improve their welfare status and to maximise their economic contribution. This systematic literature review encompasses 28 studies published between 2010–2024, that address the role of working animals and the factors impinging on their welfare. Suitability of papers for inclusion (and exclusion) involved use of a PRISMA flow diagram. In this review, we also sought to define the exact role of working animals with them found to be used not only for draught power but also as a direct source of food as well as income. A lack of medical care was also highlighted with animals afforded limited access to feed and water, subjected to regular physical abuse, and deprived of access to shelter. Insufficient assessment of welfare and improper methods of data analysis were also found to be an issue, factors that require to be addressed by future researchers to help improve the welfare of working animals in this region

## Introduction

Working animals help support the livelihood of the one billion plus people that comprise the world’s poor, playing a crucial role in helping achieve the target of the UN’s Sustainable Development Goal (SDG). Their presence enables access to education, a reduced impact of climate change, a reduction in the burden borne by women, and the delivery of water (Food and Agriculture Organisation of the United Nations [FAO] 2011; Lever & Evans 2017; Cox & Bridgers 2019; Olmos *et al.* 2021).

In terms of agriculture, working animals are responsible for more than 50% of the world’s agrarian energy with engines contributing less than 30% and the remainder provided by humans themselves (Swann 2006). Additionally, livestock are mainly considered livelihood assets and a social safety net for poor farmers, particularly for women and pastoralist farmers (Herrero *et al.* 2013; Gina *et al.* 2015).

Looking beyond agriculture, many developing parts of the world, e.g. India, Pakistan, and sub-Saharan Africa have seen urban workers shift from motor vehicles to working animals in direct response to increasing fuel prices (Haben 2020). This is the harsh reality of life in Ethiopia where working animals remain crucial, acting as the major source of income for their owners (Herago *et al.* 2015; Merridale *et al.* 2024). Despite showing variability between locations, donkeys, mules, horses and camels remain the most common transport animals in Ethiopia for pulling carts and for riding (FAO 2011; Geiger *et al*. 2020). According to the BROOKE report (2023), more than one million cart donkeys and 250,000 cart horses are utilised by millions of people throughout different parts of the country. In addition, cattle provide nearly all the draught power for agricultural crop production at the smallholder level in Ethiopia (Tefera 2011).

According to The Donkey Sanctuary Ethiopia (2017), Ethiopia has the largest working animal sector, comprising 12.4 million oxen, 5.7 million donkeys, 2.4 million camels, 2 million horses, and 0.3 million mules; animals that perform an indispensable role in the improvement of food security and helping reduce poverty. It cannot be overstated how heavily the economy of the country is reliant upon the livestock sector, contributing as it does to agricultural gross domestic product and foreign exchange as a result of exports of animals, meat, leather and other animal products (Admassu & Shiferaw 2011; Robi *et al.* 2023).

Animal welfare operates on many levels, including economic, social, political, ethical, and moral dimensions (Lund *et al.* 2006). However, there is evidence (Asebe *et al.* 2016; Gelaye 2020; Alemayehu *et al.* 2022) that despite the hugely significant role working animals play in improving livelihoods within communities and in the economy of the country as a whole, their welfare tends be neglected. There are many reasons for this. Ethiopia is still to embrace the concept of raising awareness of animals’ basic welfare requirements (Asebe *et al.* 2016; Alemayehu *et al.* 2022). For this reason, working animals become afflicted by a number of diseases, are prone to physical and mental injury, are overworked, suffer low healthcare, and see a shortage of feed, all of which result in poor welfare and work performance (Mekuria *et al.* 2013; Sumbria *et al.* 2017; Arega *et al.* 2023; Desta 2023).

Several studies have sought to explore the key health and welfare issues facing working animals in Ethiopia (Getahun *et al.* 2016; Behnke & Metaferia 2011; Shapiro *et al.* 2017; Hundie & Diba 2018; Geiger *et al* 2020; Atalel *et al.* 2024). Studies differ with regard to where their emphasis is directed, for instance Birhan *et al.* (2014), Kumar *et al.* (2014), Tesfaye *et al.* (2016), Molla *et al.* 2017, Fsahaye *et al.* (2018) and Tanga and Gebremeskel (2019) focus on the health and welfare status of working donkeys in different parts of Ethiopia whereas Tadesse (2014), Asmare and Yayeh (2017), Chala *et al.* (2017) and Merridale *et al.* (2024) were more concerned with the health and welfare status of cart-pushing horses. Meanwhile, Getnet *et al.* (2014), Ali *et al.* (2016), Solomon *et al.* (2016) and Hundie *et al.* (2018) looked at the health and welfare of cart-pulling mules. A common theme has been a fundamental lack of appreciation of the contribution made by working animals in terms of improving the livelihood and food security of smallholder farmers not to mention on the economy of Ethiopia as a whole (Shapiro *et al.* 2017; Temesgen & Sitota 2019; Geiger *et al.* 2020; Nugese 2022; Arega *et al.* 2023). Despite these studies not covering the true socio-economic contribution of working animals nor the status (or lack thereof) of their welfare, there is still merit in their findings since they focus upon a specific area in question, certain welfare parameters, and single working animals. Ultimately, what is needed is a comprehensive understanding of the welfare of working animals and the role they play in people’s livelihoods.

Thus, the main aim of this paper is to offer a systematic review of the welfare status of working animals and their role in livelihood, food security, and agriculture production in Ethiopia. In doing so, we seek to contribute in two ways; firstly, to provide comprehensive information regarding the overall welfare of working animals via an integration of previous works thereby facilitating the refining of effective strategies to improve the welfare status and economic return of working animals. And, secondly, to identify gaps in the literature as regards assessment of the welfare status of working animals. This will hopefully help inspire future researchers and policy designers regarding the welfare of working animals throughout the world.

## Materials and methods

### Literature search

For this review, the content included previously published secondary data such as books, research articles from reputable journals, annual reports from national and international organisations, policy briefs, and other indexed scholarly materials related to the topic in question. The search took place between December 2023 and April 2024.

Databases included Google Scholar, Scopus, Web of Science, and once all search sources had been identified, search criteria were refined, i.e. that studies must have been published between 2010 and 2024. Studies were limited to those incorporating such keywords as ‘working animals’, ‘equine welfare’, ‘cattle welfare’, ‘animal welfare’, ‘working animals and their role in achieving livelihood’, ‘food security’, and ‘agriculture production in Ethiopia’.

### PRISMA flow diagram description

#### Literature screening (inclusion and exclusion criteria)

**Figure 1.**
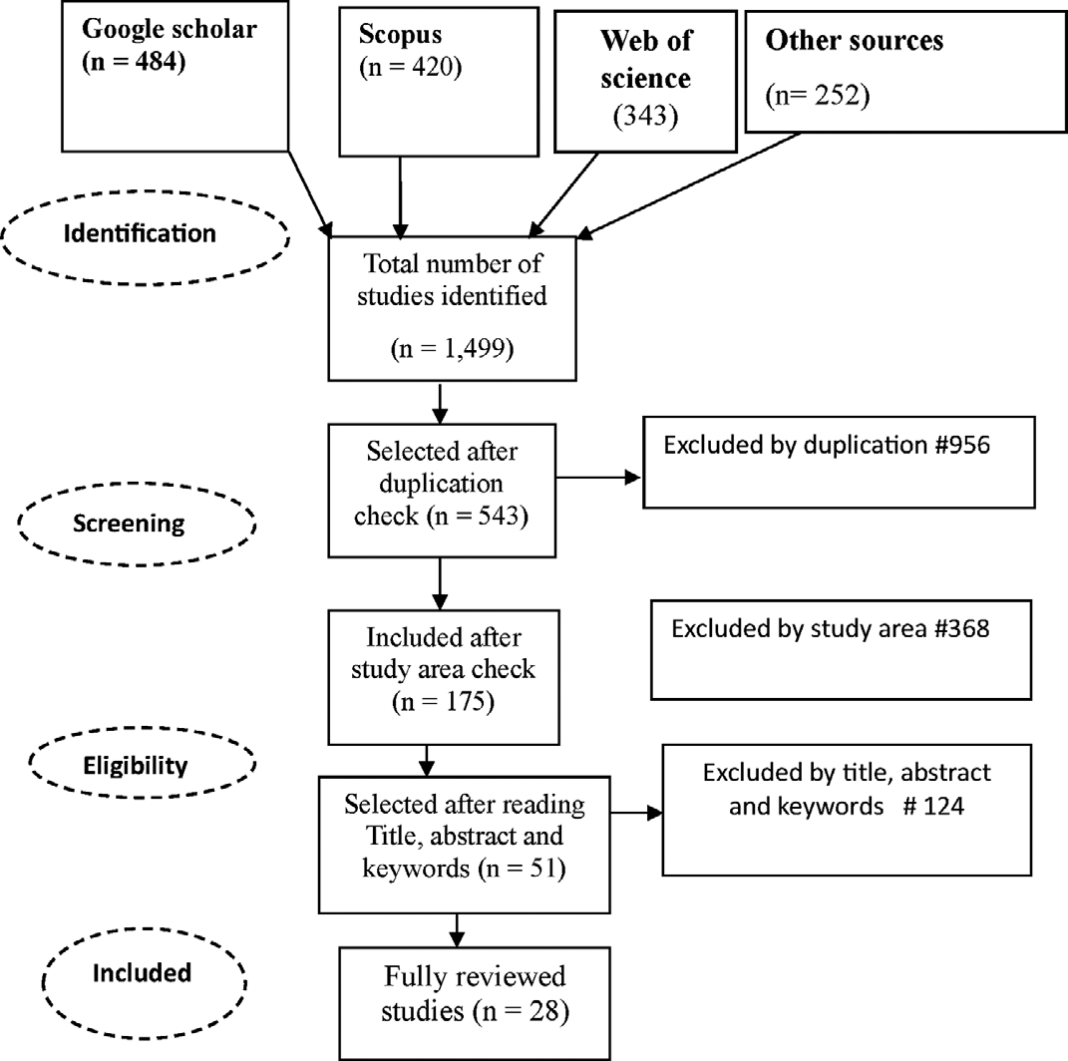
Graphic representation of the overall search and literature screening, resulting in 28 articles included in the systematic review.

The literature inclusion and exclusion criteria were developed through consideration of different principles designed to ensure literature was sourced that best achieved the review objectives. A total of 1,499 studies were initially identified which were reduced down to 28 following implementations of various inclusion and exclusion criteria. These were selected for full review. Figure 1 illustrates the entire searching and screening process, and Table 1 summarises the inclusion and exclusion criteria.

**Table 1.** Inclusion and exclusion criteria for the literature (n = 28) included in this review

Criteria	Included	Excluded	Justification for criteria
Publication date	2010 to 2024	Papers before 2010	Aim to scrutinise the recent welfare status of working animals and their economic implications
The study area (country) of the paper	Studies conducted in Ethiopia or encompassing Ethiopia	Non-Ethiopian papers	To maintain the geographical scope of the review
Publication language	Papers in English language	Papers not written in English language	To easily read and understand the papers
Publication topics	Working animals’ welfare, and their implications regarding the livelihood and food security of Ethiopians.	Papers outside working animal welfare and the subsequent implications	To attain the main aim of the systematic review
Availability of papers	Fully available papers were selected	Incomplete papers were not included	Some papers requiring a fee to be paid or not open access

## Results

### Extracted data from studies

**Figure 2.**
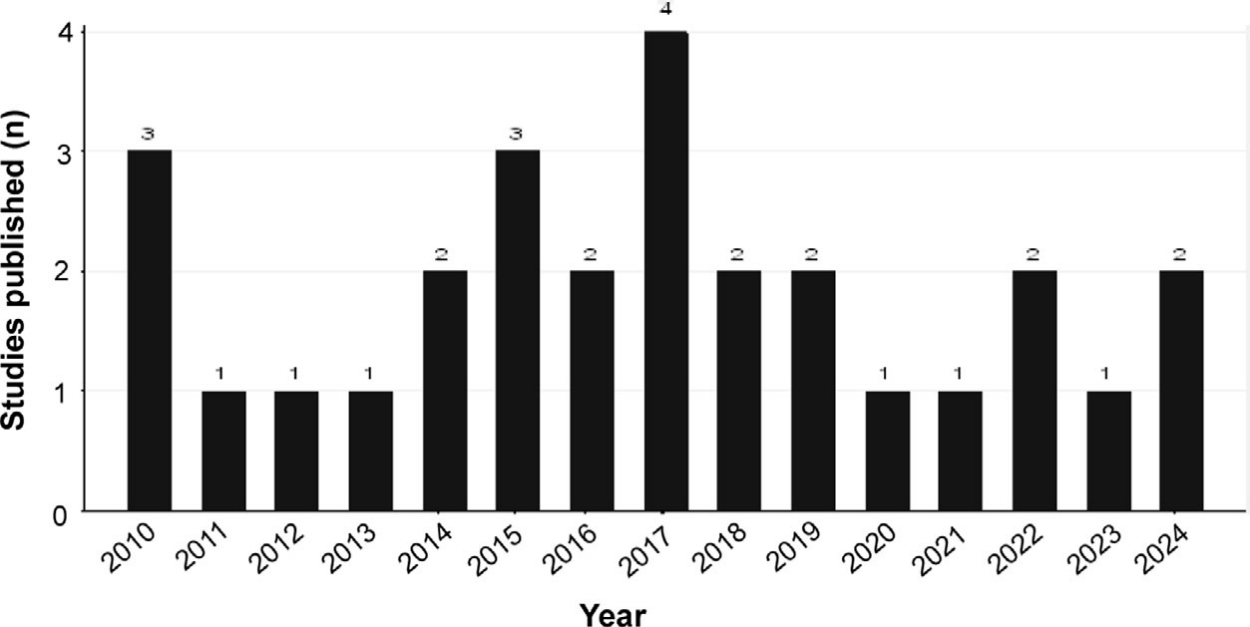
Publication years (2010–2024) of the 28 studies included in the review on working animals’ welfare and their roles in Ethiopia.

Table 2 sets out the data that were extracted and classified based on predefined criteria, including year of publication (2010–2024), study area (Ethiopia), and studies that are focused on the welfare of working animals and the role they play in people’s livelihood. The majority of selected studies addressed both working animal welfare and their role in a single study, although some looked only at the welfare aspects while others were concerned more with the economics but all were considered in this review. Figure 2 depicts the number of studies published between 2010 and 2024 on working animals’ welfare and their importance as related to livelihood and food security.

**Table 2.** Extraction of appropriate data comprising the studies (n = 28) included in review into the welfare of working animals in Ethiopia and their multidimensional implications

Author(s) and year of study	Title and study area	Working animals studied	Result of the studies	
			Role of working animals on livelihoods, food security and agriculture production	Welfare problem of Working animals
Geiger *et al.* (2020)	Understanding the attitudes of communities to the importance of working donkeys in rural and peri-urban areas of Ethiopia	Donkeys	Create economic security, independence, participation in local saving schemes, improve social status, empowerment to marginalised groups and offer a sense of companionship for poor households.	Working donkeys frequently afforded low status, are misunderstood, and given little husbandry and healthcare
Asebe *et al.* (2016)	The general status of animal welfare in developing countries: The case of Ethiopia	Equines and cattle	Not addressed	Less concern about animal welfare and no comprehensive legislation, rules or regulations formulated to protect animals
Tanga & Gebremeskel (2019)	The neglected welfare state of working donkeys in Ethiopia: The case of Dale district in Southern Ethiopia	Donkeys	Not addressed	Feed shortage, poor health, low social status and low income of owners
Admassu & Shiferaw (2011)	Donkeys, horses and mules: Their contribution to people’s livelihoods in Ethiopia	Donkeys, horses and mules	Creation of employment opportunities (gharry and cart services), access to finance (renting equines), local transportation and other livelihood activities	Feed shortage, poor health, low social status and poor management
Amante *et al.* (2014)	Health and welfare assessment of working equines in and around Nekemte Town, East Wollega Zone, Ethiopia	Equines (horses donkeys mules)	Not addressed	Shortage of feed. Healthcare dealt with traditionally. Wound problems, overworking, overloading, issues with housing
Cochet (2011)	A new perspective on animal traction in Ethiopian agriculture	Oxen	Oxen play a significant role as a draught power for crop production	Not addressed
Alemayehu *et al.* (2022)	Animal welfare knowledge, attitudes, and practices among livestock holders in Ethiopia	Cattle and equines	Not addressed	Poor management, poor nutrition, lack of disease prevention and medical treatment. No responsible care of animals nor humane handling
Mouazen *et al.* (2007)	Improving animal drawn tillage systems in the Ethiopian highlands	Oxen	Oxen are the main source of draught power in crop production	Not addressed
Asmare & Yayeh (2017)	Assessment on the management of draft horses in selected areas of Awi Zone, Ethiopia	Horses	Horses used for ploughing, packing goods and riding for human transport and play role in livelihood improvement	Lack of grazing land, lack of training and knowledge on horse feeding management system and disease
Arega *et al.* (2023)	Welfare problems of equines in Sebeta Town and suburbs, central Ethiopia	Equines	Equines play significant role in transportation people and goods	Excessive workload in conjunction with insufficient feed, water, and veterinary treatment
Aune *et al.* (2001)	The ox ploughing system in Ethiopia: can it be sustained	Oxen	Oxen used for traction of farming land and crop threshing	Not addressed
Molla *et al.* (2021)	Estimating the economic impact and assessing epizootic lymphangitis in equines in Gondar Zones, Ethiopia	Equines	Equines play an indispensable role in helping sustain cart businesses as alternative sources of income and means of survival for many households to attain their food security	Equine owners with insufficient knowledge, poor control and lack of disease prevention
Herago *et al.* (2015)	Assessment on working donkey welfare issue in Wolaita Soddo Zuria district, southern Ethiopia	Donkeys	Not addressed	Poor healthcare, overworking and overloading
Birhan *et al.* (2014)	Incidence of wound and associated risk factors in working donkeys in Yilmana Densa District	Donkeys	Not addressed	Greater prevalence of wounds in older, adult donkeys than young
Molla *et al.* (2017)	The welfare, watering, housing, feeding and working management of working donkeys in and around Hawassa City, Southern Ethiopia	Donkeys	Not addressed	Skin problems, behavioural issues, external wounds and sores
Chala *et al.* (2017)	Prevalence of work-related wounds and the associated risk factors in cart horses in Bishoftu Town, Central Ethiopia	Horses	Income source via transport services, employment creation for poor households and youths	Wounds, improper harnesses and saddle, poor breast strap, poor girth rope and infectious diseases
Nejash *et al.* (2017)	Prevalence and associated risk factors of equine wound in and around Asella town, South Eastern Ethiopia	All equines	Not addressed	Problems with harnesses and infectious diseases
Tesfaye *et al.* (2016)	Study on the health and welfare of working donkeys in Mirab Abaya District, Southern Ethiopia	Donkeys	For transportation of commodities, agricultural input and outputs	Musculoskeletal problems, dermatological diseases, eye and dental problems
Ayalew *et al.* (2018)	Monitoring of body weight, body condition and observation of wound on working equines in Ethiopia	All equines	Not addressed	Injury, improper harnesses, over-working
Mekete (2022)	The prevalence of work-related wound and associated risk factors in working equines	All equines	Not addressed	Prevalence of external injuries
Mekuria & Abebe (2013)	Observation on major welfare problems of equines in Meskan district, Southern Ethiopia	All equines	Not addressed	Limited feed, water and shelter
Usman*et al.* (2015)	Health and welfare related assessment of working equines in and around Batu Town, East Shoa, Central Ethiopia	Equines	Not addressed	Problems with harnesses, overloading, wounds, overworking, disease, lack of veterinary care, and insufficient feed
Mekonnen & Channe (2016)	Management practices of working donkeys in urban and rural areas of Assosa District, Benishangul gumuze region, Ethiopia	Donkeys	Assisting livelihood via transportation of people, working as pack materials and serving as a source of income for poor farmers	Feeding, housing, health and sanitation problems
Girmay (2017)	Health and welfare assessment of working donkeys in and around Axum Districts, Tigray Regional State Northern Ethiopia	Donkeys	Donkeys play an important role in the transportation of farm produce from the field to home and the market	Wounds, no treatment, no water or appropriate shelter
Yalew *et al.* (2024)	Assessment of community-based intervention approaches to improve the health and welfare of working donkeys region, Southern Ethiopia	Donkeys	Not addressed	Prevalence and severity of lameness and wounds in donkeys
Fsahaye *et al.* (2018)	Health and welfare assessment of working donkeys in and around Rama town, Tigray, Ethiopia	Donkeys	Donkeys provide draught power in crop production, low cost transport and are a source of income	Presence of wounds, parasitic and behavioural problems
Chalchisa *et al.* (2024)	Biting flies and associated pathogens in camels in Amibara District of Afar Region, Ethiopia	Camels only	Not addressed	*Hippobosca* and *Stomoxys* are biting flies that affect camels’ health and body condition
Gina *et al.* (2015)	The role of working animals toward livelihoods and food security in selected districts of Fafan Zone, Somali Region, Ethiopia	Camels, equines and cattle	They play a vital role as a source of income, food and as draught power for different activities	Not addressed

Our review found 53% of studies that stressed the importance of working animals as draught labour, while 36% indicated the critical role of working animals as a primary source of income and employment creation especially for poor people in rural and urban areas. A further 7% described the role of working animals as direct sources of food.

## Discussion

### The major welfare problems facing working animals in Ethiopia

Prior to any discussion regarding the welfare status of these working animals it might be helpful to consider the basic indicators of farm animal welfare. The Farm Animal Welfare Committee (FAWC 2011) sets out five standardised welfare indicators for the welfare status of animals. The so-called Five Freedoms which consist of ‘*Freedom from hunger and thirst*’, ‘*Freedom from discomfort’*, ‘*Freedom from pain, injury, or disease’*, ‘*Freedom to express normal behaviour’* and ‘*Freedom from fear and distress’.* These are worth bearing in mind when viewed in conjunction with Table 3 which shows the main working animal species in Ethiopia, the major welfare problems facing each animal and the solution recommended in previous studies.

**Table 3.** Summary of the findings from the studies (n = 28) included in our review, sorted by species of working animal with their predominant welfare problems and potential solutions

Working animals in Ethiopia	Predominant welfare problems for each working animal	Recommended solutions to reduce welfare problems	References
Donkeys	Improper harnesses Skin abrasions, wounds, musculo-skeletal, parasitic, ocular and behavioural problems Poor animal handling practices and insufficient attention paid to donkeys Feed and water shortages, Limited veterinary support	Raising awareness of donkey welfare to users Context-specific welfare improvement interventions. Training and supplementary care service provided to donkey owners	Lemma *et al.* (2022), Fsahaye *et al.* (2018) Herago *et al.* (2015)
Mules	External injuries caused by problems with harnesses Insufficient care and attention provided, compared to other farm animals Acute lameness	Community education on injury mitigation schemes Extensive awareness of welfare issues for cart mule owners and drivers Improvement to modern health-seeking behaviour of owners Replacement of poorly designed harness and accessories	Getnet *et al.* (2014), Ali *et al.* (2016). Amante *et al.* (2014).
Horses	Feed shortage, poor health care, lameness, wound abrasions, overworking, overloading, issues with housing	Raising awareness of animal welfare within rural communities Farmers need to be educated on horse husbandry and welfare -	Fasil & Yenewhunegnaw (2017)
Oxen	Working in stressful conditions deprived of feed and water Feeding disorders, including bloating Suffer from wounds as a result of whipping, beating and poking, and infections and bruising beneath the yoke	Improve nutrition, healthcare, breeding and working conditions Raising farmers’ awareness of health issues that arise from improper use of oxen	Bobobee & Gebresenbet (2007), Tefera (2011). Urga & Abayneh (2007).
Camels	Affected by various diseases (*Cephalopina titillator* larvae, camel contagious ecthyma [CCE]) Overloading, lack of feed and water and general neglect from owners Diseases, poor veterinary care and lack of attention from government.	Strategic community education to potentially improve camel management systems Encourage disease control and intervention	Teha *et al.* (2017) Melkamu *et al.* (2022). Nuguse (2022), Previti *et al.* (2016).

**Figure 3.**
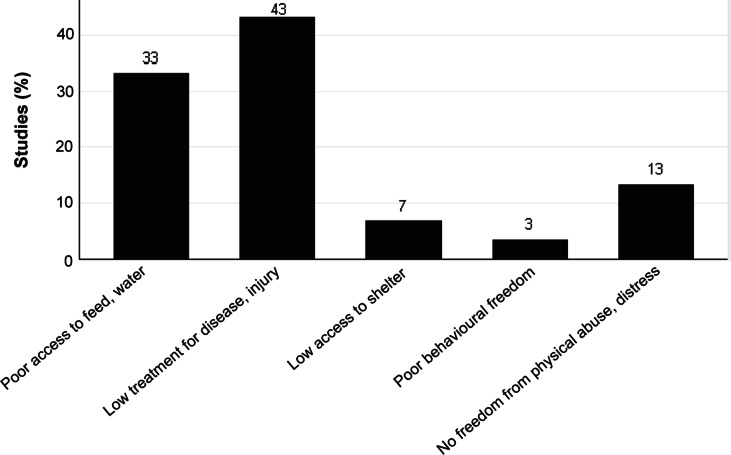
Major welfare problems facing working animals in Ethiopia according to the 28 studies included in the review.

Figure 3 offers an illustration of the major welfare problems faced by working animals as reported in previous studies published from 2010–2024. Our review revealed 43% of studies specified low prevention and treatment of injury or disease as a primary welfare problem for working animals in Ethiopia. Despite the extensive roles played by equines in the community, less attention gets paid to their welfare (Upjohn *et al.* 2014; Geiger *et al.* 2021; Arega *et al.* 2023). It is clear from our review that the majority of owners do not take their equines to veterinary clinics for treatment with some relying upon traditional medicines. The picture painted is one where equines were expected to work hard without sufficient veterinary treatment and when sick they are essentially left to die (Zegeye *et al.* 2015; Stringer *et al.* 2017).

There is extensive literature on the subject of working animals’ susceptibility to health-related issues, such as infectious diseases, dermatological diseases, dental problems, musculoskeletal problems, eye problems, wounds, abrasions from ill-fitting breast straps, poor girth, improper harnessing, and biting insects (*Hippobosca* spp, *Stomoxys* spp) as direct result of their limited access to veterinary treatment (Lund *et al.* 2006; Birhan *et al.* 2014; Tesfaye *et al.* 2016; Nejash *et al*. 2017; Fsahaye *et al.* 2018; Melkamu *et al.* 2022; Mekete 2022; Chalchisa *et al.* 2024).

*Our* review found that 33% of Ethiopa’s working animals suffered from limited access to feed and water. Equines, especially donkeys, are deemed low status animals throughout different regions of Ethiopia and, as such, are frequently neglected; denied access the kind of feed, water, and healthcare that is made readily available to other animals such as cattle. An outcome implying that equines, despite the fundamental role they play in socio-economic enterprise, are perceived as being of little value by their owners (Admassu & Shiferaw 2011; Amante *et al.* 2014; Asebe *et al.* 2016; Tanga & Gebremeskel 2019; Geiger *et al.* 2020; Alemayehu *et al.* 2022)

Our analysis also found evidence that in 13% of the studies reviewed, working animals had no freedom from beating, fear, and distress. Many of the working equines are owned by poor households; hence, ensuring conditions and treatments that avoid mental and physical suffering are ignored (Teferi *et al.* 2020; Lemma *et al.* 2022). Equines are beaten to compel them to work and, as a result, are subject to serious ailments, including gait abnormalities, joint swelling, broken skin, deep lesions, and dental problems (Birhan *et al.* 2014; Ayalew *et al.* 2018; Temesgen & Sitota 2019; Alemayehu *et al.* 2022; Mekete 2022).

As shown in Figure 3, 7% of papers reviewed reported working animals, particularly equines, were provided insufficient access to shelter and resting areas (Usman *et al.* 2015; Mekonnen & Channe 2016; Girmay 2017; Fsahaye *et al.* 2018; Yalew *et al.* 2024). This indicates the seriousness of this issue for working animals (especially equines) in Ethiopia. Since cattle are held in higher status (as a result of their meat being a prized commodity), mostly they are provided with access to shelter. The majority of farmers utilise cattle for a dual purpose; firstly, as a traction force and then latterly the meat is sold as a source of food. This serves as a direct contrast to equines since their meat is not deemed consumable within communities leading to them suffering neglect (Amante *et al.* 2014; Geiger *et al.* 2020; Aliye *et al.* 2022).

Improving the conditions and facilities for working animals to enable them to express their normal behaviour is a key indicator of welfare (Popescu & Diugan 2013; Ali *et al.* 2016). As shown in Figure 3, only 3% of the studies reviewed conducted behavioural assessments on working equines in Ethiopia thereby ensuring a distinct lack of possibilities for equines to express their normal behaviour. A significant number of studies indicate the importance of the human-animal relationship to working animals’ care as well as for effective utilisation of them, but mostly the studies express the notion that the majority of working animals are dull and difficult to catch leading to them being beaten and suffering from chronic fear (Usman *et al.* 2015; Getahun *et al.* 2016; Fsahaye *et al.* 2018; Yalew *et al.* 2024).

### The role working animals play in improving livelihood, food security and agriculture production

Ethiopia has a reported population of 31 million cattle which is the largest in the entire continent of Africa (Astatke & Mohammed-Saleem 1996; Aune *et al.* 2001). Nine to 10 million of which are deployed as draught power in agriculture or for other livelihood purposes. Typically, Ethiopian smallholder farms make use of indigenous breeds of Zebu cattle, deployed in pairs, to prepare seed beds and for threshing. Horses, donkeys and mules tend only to be used to plough crop land when oxen are unavailable when they are paired with the same species or with others (Taye 2005; Urga & Abayneh 2007; Chanie *et al.* 2012). Generally, these equine species (horses, donkeys and mules) are deployed as modes of transport throughout most parts of the country. Ethiopia has six donkey breeds (Abyssinian, Afar, Hararghe, Ogaden Omo and Sinnar) which are distributed throughout the highland region (Gebreab *et al.* 2004; Zewdie *et al.* 2015; Getachew *et al.* 2023). In Ethiopia, donkeys play a significant role in transport, pulling carts and for ploughing (Central Statistical Agency [CSA] 2017; Wassie *et al.* 2023). In the lowlands and in drier regions, camels are also used exclusively as pack and transport animals.

Several scholars (Teha *et al*. 2017; Wilson 2010; Admassu & Shiferaw 2011; Girmay 2017) confirmed the broad use of working animals throughout Ethiopia and the indispensable role they play in people’s everyday activities, not to mention the income that they also generate. Furthermore, they have both direct and indirect impact on households’ overall standard of living through helping ensure food will be available.

This was illustrated in a study by Amejo *et al.* (2018) who described cattle as not only a direct source of food via provision of meat and milk but also an indirect source due to their role as draught animals, ploughing, threshing and, on occasion, as transporters of products. In Ethiopia, 90% of the rural population are dependent upon draught animals, including oxen, for a multitude of purposes. A number of other studies (Li *et al.* 2023; Wendimu *et al.* 2023; Willy *et al.* 2023; Berhe *et al.* 2024) indicate oxen as being important sources of income via direct sales of animals, meat, skin, and other products. Oxen may also be rented out for cash during the cropping season, creating further income for farmers (Mouazen *et al.* 2007; Cochet 2011). According to Aune *et al.* (2001), oxen are the primary source of draught power for crop production, however in rare circumstances farmers will also use cow traction as an alternative to oxen ploughing. However, the deployment of cows as a traction power was not referred to in the papers covered in our systematic review. The majority of farmers in Ethiopia use oxen as traction for ploughing their land for agricultural crop production. Farmers also use cattle production as an alternative source of income by selling them for meat at the local market. However, welfare problems afflicting oxen during the ploughing season has a detrimental effect on their meat quality leading to a decline in their market price and reduced profit margin (Jemal *et al.* 2023).

Our review also highlighted the role played by camels, with them used as draught power and transporters of both people and products across landscapes less suited to other working animals. Additionally, they also serve as a reliable food source in areas of Ethiopia where the arid and semi-arid conditions render other animals less practical. Although the consumption of camel meat and milk is frowned upon by the Christian church, these remain an important food source for the pastoral and agro-pastoral Muslim communities of Ethiopia (Mouazen *et al.* 2007; Tura *et al.* 2010; Demlie *et al.* 2023; Edea *et al.* 2024).

Further evidence, originating from the Somali Region of Ethiopia, vindicates this notion of working animals (e.g. camels, donkeys and cattle) contributing greatly to household finances as well as enriching the lives of the community in general. Cattle and camels especially represent basic and reliable sources of meat, milk, and skin for households (Gina *et al*. 2015). In line with the above study, the FAO (2011) also reported the direct role played by working animals in ensuring food availability at the level of the smallholder farmers via provision of milk and meat and indirectly as a result of fertilisation of farmland and the sale of offspring to fund food items. For developing countries such as Ethiopia, in particular, the majority of the population depends on animal power as its main energy source.

Our review also sheds light on the role equines play in creating employment opportunities for poor households, leading to the generation of income. The majority of equine owners confirmed that the benefits far outweigh the costs as regards equines, making them very useful either for use exclusively by the household or for the generation of income. As the majority of Ethiopians depend greatly upon subsistence agriculture, which is highly susceptible to climatic change risk, our study has identified that equines are the main source for diversification into non-farm activities (Ameni 2006; Admassu & Shiferaw 2011; Gelaye 2020; Molla *et al.* 2021). In Ethiopia, especially the study areas of Ankesha Guagusa and Banja Shekudad district, evidence points to the importance of equines, such as horses, to the livelihood of many smallholder farmers. For example, ploughing of farmland, transportation of goods, and human transport (Asmare & Yayeh 2017).

### Research gaps

Previously, studies have sought to investigate the overall welfare of working animals and the roles they play regarding livelihood, food security and agricultural production. Seeking to diminish welfare issues as well as enhancing the impact of working animals on food security is key, however additional efforts must also be devoted to advancing research in this field. As such, this systematic review has set out to identify research gaps in existing literature.

#### Gaps in the measurement of working animals’ welfare

Applying accurate welfare indicators (measurements) or parameters is a necessary precondition in assessing and understanding the welfare status of working animals. Only a very small proportion of the total papers reviewed considered all the parameters in one single investigation, e.g. Girmay (2017), Tanga and Gebremeskel (2019), Aliye *et al.* (2022) and Yalew *et al.* (2024). Meanwhile, 40% of studies reviewed focused only on the health and physical condition of the animals in question, e.g. Scantlebury *et al.* (2015), Fasil and Yenewhunegnaw (2017), Fsahaye *et al.* (2018), Molla *et al.* (2021) and Mathewos *et al.* (2023). On the other hand, 37% of studies were concerned with feed and water access for working animals, e.g. Amenu *et al.* (2013), Asmare and Yayeh (2017) and Hadush (2018). However, tellingly, only 3% (Asmare 2014; Molla *et al.* 2017) looked at the behaviour of working animals and their mental state. This indicates a need for studies to consider a holistic approach whereby the overall welfare status of working animals is considered, not merely one facet. Clearly, there has been a failure to pay sufficient attention to this with health parameters mostly featuring in assessments of working animals’ welfare.

#### Gaps in the data analysis methods

According to our analysis, 46.6% made use of simple descriptive statistics to analyse their data. Meanwhile only 27% of studies deployed econometric models with 20% and 7% using narration and other methods of analysis, respectively. Despite selecting a method of data analysis appropriate to the study in question, econometric models are preferable due to their ability to identify the relevance and predict the magnitude of the specific welfare parameter in question, thereby improving the welfare status of the working animals. Unfortunately, little attention has been paid to either econometrics or other sophisticated methods of analysis in these studies.

#### Gap in studied working animals

The largest proportion of previous studies (43%) were focused purely on donkeys, whereas 30% took all equines (donkeys, horses, and mules) into account. Moreover, 13% of studies considered only horses, while 7% were concerned with both cattle and equines. Further statistics to emerge were that only 3% focused on cattle and the same proportion on camels. Cattle and camels are among the most vital working animals in Ethiopia (Aune *et al.* 2001; Gina *et al.* 2015) yet, despite this, remain the subject of a paucity of studies in comparison to working equines.

## Animal welfare implications and conclusion

The main aim of this review was to demonstrate the role of working animals in people’s livelihoods, food security, farm production, and poverty reduction in an attempt to increase the profile of these animals among policy-makers, thereby enhancing the implementation of policies to secure working animal welfare. Our findings highlight the close connection that exists between human livelihoods, food security, agricultural production, and working animals. However, the welfare of working animals in developing countries such as Ethiopia continues to be a cause for concern. To this end, we carried out a literature review of papers published between 2010 and 2024. The results revealed the multi-dimensional importance of working animals to be encapsulated into a number of major roles, including serving as a draught power for the majority of smallholder farmers, as a basic source of income, as a direct source of food, not to mention other social and cultural roles. Numerous welfare issues were readily identified that seriously impinge on the ability of these animals to work efficiently. These mainly consisted of high occurrence of disease and injury in conjunction with low to non-existent treatment/veterinary care; poor access to feed and water, lack of freedom from beatings and distress; low access to shelter; and no freedom to express normal behaviour. In addition, a number of research gaps were identified as regards aspects of welfare measurements and methods of data analysis which led us to offer a set of recommendations to be addressed moving forward.

In terms of the challenge of meeting the needs of food supply, working animals can act as a fundamental sustainable food resource, for human livelihoods, and reduction of poverty in Ethiopia. This could influence policy-makers and development partners to incorporate these animals into the national development plans, as part of a holistic approach to help ensure food security and poverty reduction. In particular, all bodies in question should prioritise the need to popularise and maintain the animal welfare protection system from national to ground level. Moreover, future studies should strive to investigate the status of animal welfare via an integrated and holistic approach, ensuring the implementation of appropriate welfare indicators and rigorous data analysis methods.
